# Does replication groups scoring reduce false positive rate in SNP interaction discovery?

**DOI:** 10.1186/1471-2164-11-58

**Published:** 2010-01-22

**Authors:** Marko Toplak, Tomaz Curk, Janez Demsar, Blaz Zupan

**Affiliations:** 1 Faculty of Computer and Information Science, University of Ljubljana, Tržaška 25, SI-1000 Ljubljana, Slovenia; 2 Department of Molecular and Human Genetics, Baylor College of Medicine, 1 Baylor Plaza, Houston, TX 77030, USA

## Abstract

**Background:**

Computational methods that infer single nucleotide polymorphism (SNP) interactions from phenotype data may uncover new biological mechanisms in non-Mendelian diseases. However, practical aspects of such analysis face many problems. Present experimental studies typically use SNP arrays with hundreds of thousands of SNPs but record only hundreds of samples. Candidate SNP pairs inferred by interaction analysis may include a high proportion of false positives. Recently, Gayan et al. (2008) proposed to reduce the number of false positives by combining results of interaction analysis performed on subsets of data (replication groups), rather than analyzing the entire data set directly. If performing as hypothesized, replication groups scoring could improve interaction analysis and also any type of feature ranking and selection procedure in systems biology. Because Gayan et al. do not compare their approach to the standard interaction analysis techniques, we here investigate if replication groups indeed reduce the number of reported false positive interactions.

**Results:**

A set of simulated and false interaction-imputed experimental SNP data sets were used to compare the inference of SNP-SNP interactions by means of replication groups to the standard approach where the entire data set was directly used to score all candidate SNP pairs. In all our experiments, the inference of interactions from the entire data set (e.g. without using the replication groups) reported fewer false positives.

**Conclusions:**

With respect to the direct scoring approach the utility of replication groups does not reduce false positive rates, and may, depending on the data set, often perform worse.

## Background

Onsets of many common chronic diseases are governed by genetic factors that do not follow "Mendelian" or "single gene" patterns. Such diseases include hypertension, diabetes, various cancers, Alzheimer's disease, heart disease, Parkinson's disease, and others. Genetics governing the susceptibility to these diseases remains largely unknown. Their onset may be triggered by polymorphisms across the genome whose effects do not simply (linearly) sum up but instead interact in complex, non-linear fashion. Such interactions are also referred to as *epistasis *[1].

A number of computational methods for detection of epistasis of single nucleotide polymorphisms (SNPs) have been proposed [2]. They can be based either on regression models [3], data mining [4], goodness of fit tests [5] or information theory [6,7]. These methods consider data sets that include phenotype observations (presence or absence of a disease) in several hundreds to several thousands cases and controls, each characterized by a whole-genome profile consisting of several hundred thousands SNPs. Synergistic SNPs may in the extreme provide no information on the disease by themselves, so the search for interesting SNP-SNP interactions needs to consider all candidate pairs. In a study using SNP chips with a million probes, analysis of epistasis requires scoring of about 5·10^11 ^hypotheses - one for each candidate pair. Due to limited number of samples, the number of spurious false positive results can be overwhelming.

To reduce the number of reported false positive interactions, Gayan et al. (2008) have recently proposed a scoring approach called Hypothesis Free Clinical Cloning (HFCC). The part of HFCC used for interaction scoring is based on so-called *replication groups*, which splits the available samples into non-overlapping subsets, and reports only on SNP interactions with minimal interaction score across all subsets above a certain threshold. Authors hypothesize that this approach may allow identification of frequent and consistent epistatic effects at the expense of lower test power, improving the filtering of false positive results at the expense of increasing false negative rate.

Gayan et al. demonstrate the utility of HFCC in a practical application, but do not specifically address their otherwise intuitive assertion on the reduction of false positive rate by HFCC. We were curious if the utility of replication groups indeed performs as suggested. Namely, if so, the approach would not only advance the field of epistasis analysis, but could also spark new improvements in techniques for SNP, gene and protein scoring and ranking, where standard feature selection procedures face similar problems due to low samples-to-features rate.

We compared the SNP interaction scoring with replication groups to the standard procedure which uses the entire data set. We performed experiments on simulated data and five data sets from Gene expression Omnibus (GEO) [8]. We were unable to confirm that the use of replication groups reduces the number of false positive results. On the contrary, the standard approach performed better in all our experiments.

## Data

We performed the evaluation on simulated data, where false positive interactions are known, and on false interaction-imputed GEO data sets.

### Simulated data sets

**Figure 1:**
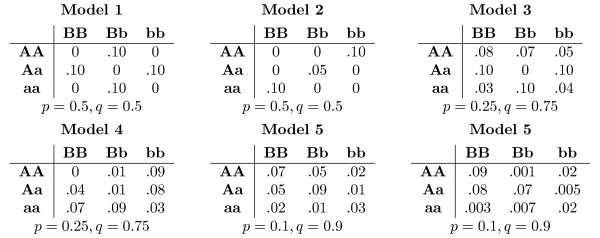
**Disease penetrance models**. Penetrance models used to simulate epistasis between two SNPs. Allele frequencies are denoted with *p *and *q*. For example, model 1 specifies that 10% of individuals with genotypes AABb, AaBB, Aabb or aaBb and none of individuals with other genotypes have the disease.

We followed the data synthesis protocol as proposed by Ritchie et al. (2003). The simulated data sets were generated according to six two-SNP epistasis models (see Figure 1). Unlike Ritchie et al. (2003), our data sets included multiple interactions, but such that each SNP was involved in interaction with at most one other SNP. Two different types of data sets with respect to the number of SNPs were crafted, each comprising 200 control and 200 disease samples:

1. data sets with 100 SNPs (syn1), where each data set included 24 SNP interactions (four interactions for each of six epistasis models),

2. data sets with 500 SNPs (syn2), where each data set included 60 SNP interactions (ten interactions for each model).

Several simulated data sets were subject to different types of noise including missing data (mN), genotyping error (gN), phenocopies (pN), and genetic heterogeneity (hN). Noise was imputed according to methods described by Ritchie et al. (2003). Throughout this report, data set names indicate the number of SNPs (syn1 or syn2) and the type of the noise used (either no suffix where no noise was applied, noise type where a single type of noise was applied, or AN where all types of noise were applied simultaneously).

### SNP data from Gene Expression Omnibus

Gene Expression Omnibus [8] was considered for SNP data sets that contain at least 200 samples with approximately equal case/control distribution. Five data sets met these criteria:

• GSE6754 [9] describing families with two individuals affected by autism spectrum disorders. Individuals were classified as affected (2459 samples) or unaffected (3473 samples) and described with around 10,000 SNPs each.

• GSE8054 [10] comprising 901 SNPs for each of the 121 cancerous samples and 87 controls.

• GSE8055 [10] comprising 1,189 SNPs for each of the 141 cancerous samples and 89 controls.

• GSE7226 [11] with platform designation GPL2004, comprising around 50,000 SNPs for each of the 102 samples from mentally retarded children and 213 controls from their unaffected siblings or parents.

• GSE7226 [11] with platform designation GPL2005, comprising around 50,000 SNPs for each of the 103 samples from mentally retarded children and 210 controls from their unaffected siblings or parents.

True and false interactions in these data sets are in general unknown. To enable our evaluation, we have destroyed any potential interaction for one half of the SNPs by randomly permuting their values across the samples. Pairs which include permuted SNPs are considered as false positives when chosen by the epistasis analysis method.

## Methods

### Interaction analysis

Let *X *and *Y *be a pair of SNPs and *S *a data set that includes these two SNPs and records the phenotype observations. Let *f*^*S*^(*X*, *Y*) be an interaction score, that is, the degree of synergy between SNPs *X *and *Y *when these two combined are used to predict the phenotype. In our study, we use two different measures of synergy: a measure that is a part of HFCC program suite [5], and a estimate based on information theory called *interaction gain *[6,7]. In short, HFCC considers a set of two-SNP disease models [12] and for each assesses how likely these fit the data. The model with the best fit is used to compute the interaction score of a pair of SNPs. The interaction gain approach estimates information gained by considering the two SNPs together as compared to when they are considered separately [6].

**Figure 2:**
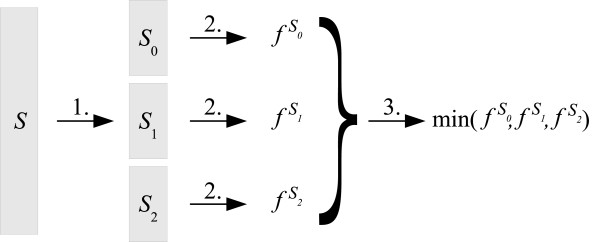
**Replication groups scoring**. Replication groups scoring involves three steps: (1) data partitioning, (2) assessment of score (*X*, *Y*) for a given SNP pair (*X*, *Y*) on a replication group *S*_*i*_, and (3) computation of the final score min_0≤*i*<*r *_(*X*, *Y*).

In our study we compare the approach where the measures of synergy are computed from the entire data set to the replication group-based approach, which estimates the synergies from data subsets and then returns the minimal score. SNP interaction scoring by replication groups proceeds as follows (Figure 2):

1. Randomly split the samples in the data set *S *into *r *disjunct sets *S*_*i *_of approximately equal sizes and class distributions.

2. Compute an interaction score (*X*, *Y*) for each SNP pair (*X*, *Y*) and for each of the subsets *S*_*i*_.

3. Given a threshold *T*, SNP pair (X, Y) is relevant if (*X*, *Y*) > *T *for all 0 ≤ *i *<*r *or, equivalently, SNP pair (*X*, *Y*) is relevant if min_0≤*i*<*r *_(*X*, *Y*) > *T*.

For the purpose of ranking, replication groups scoring assigns the SNP interaction score equal to min_0≤*i*<*r *_(*X*, *Y*), that is, the minimal score across the *r *data subsets.

We used the binary version of HFCC software provided as a supplement to Gayan et al. (2008), performing exhaustive two-locus searches with nine simple disease models that, as in the original article, include only one high-risk two-locus combination. Due to explicitly imposed limitations of this freely-available software we could only analyze data sets with fewer than 600 samples. In this implementation, the size of the file holding intermediate results of HFCC can not exceed 2 gigabytes; as we were interested in ranking of entire set of SNP pairs, this limited our studies to about 2,000 SNPs. Therefore we only considered the first 2,000 SNPs of each data set, and a stratified sample of 500 individuals was used for the GSE6754 data set.

### Experimental methodology

Feature scoring assigns interaction scores to all pairs of SNPs, resulting in a ranked list of SNP pairs. Either pairs of SNPs with scores exceeding a certain threshold or a fixed number of top-scored pairs are usually reported. As these thresholds are in general unknown, we considered sets of *k *best candidate interactions, where *k *ranged from 1 to the number of all SNP pairs. We report the results graphically, showing the dependency of false positive count on the number of considered best-scored SNP pairs. In addition to using three replication groups, as proposed in Gayan et al. (2008), we also performed experiments with two replication groups. To discount random variation in observed quality scores, we report performance score averages across 50 bootstrap samples for experimental data sets. Similarly, the results for simulated data sets are averaged across 100 repetitions of data sets created using different random seeds.

## Results and discussion

**Figure 3:**
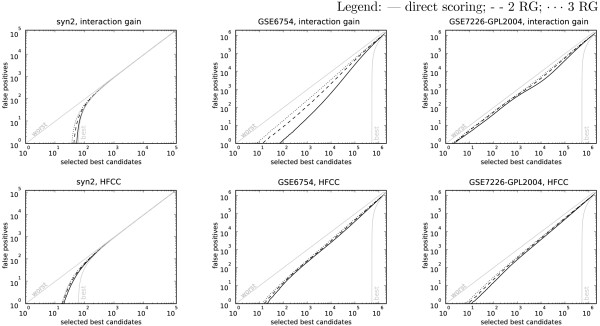
**Performance graphs**. The dependency of false positive counts given the number of selected best candidate interactions. A direct scoring (solid curves) was compared to scoring with two (dashed curves) or three (dotted curves) replication groups (RG). Curves closer to lower-right corner of the graph indicate better performance. The axes are in logarithmic scale to emphasize the results for smaller numbers of best candidates. The theoretically best and worst possible performance curves are shown in light gray.

Figure 3 presents typical results on three different data sets, where SNP interactions are scored either using HFCC or interaction gain. In all three data sets, independently of interaction scoring method, the replication groups scoring increased the proportion of false positives. Similar was found in all other data sets (see Additional file 1 for performance graphs on other data sets). That is, consistently across our array of experiments, scoring SNP interactions directly from the entire data sets performed better than replication groups scoring with either two or three replications.

Replication groups scoring uses non-overlapping data samples. With three groups, the scoring technique considers samples of one third of the original data set size. This undersampling may be the main reason for consistently better performance of direct scoring. Better performance of replication groups scoring that uses two groups instead of three supports this hypothesis. To investigate this further, we additionally experimented with bootstrap sampling (sampling with replacement) and used it instead of data partitioning. The results (Additional file 2) show improved performance against replication groups scoring, and similar performance as the direct scoring, especially with increased number of bootstrap samples. Direct scoring performed consistently better than bootstrap sampling in all simulated data sets, and better in two out of five data sets from GEO.

An alternative hypothesis for poor performance of replication groups scoring is the low number of samples in the data sets. Results using the interaction gain (Additional file 3) show that increasing the number of samples reduces false positive rate for both the direct approach and replication groups scoring, with the direct approach still performing consistently better.

From the viewpoint of statistics, we are trying to rank the interactions based on the HFCC score or another measure of interaction. To estimate the true score from a sample data, we can use either a direct approach or compute the score on multiple subsets of the data and aggregate them in some way. The usual aggregation method is computing the average score; such aggregates are conveniently distributed normally around the true value, according to the central limit theorem. Replication groups scoring aggregates by taking the minimal interaction score. Minima are not governed by the central limit theorem but are instead distributed by the Gumbel's extreme value distribution, which depends on the number of subsets (2 or 3 in Gayan's paper, more in bootstrap) and the shape of the distribution of the score. Minima are not estimators of the true value, yet the ranking of interactions by minima could correspond to the underlying unknown true ranking. As we showed, this is mostly not the case, except in some experiments with bootstrap sampling. The reasons for the apparent success of bootstrap in those particular cases are difficult to explain and may be a random fluke or they may indicate that the distribution of the observed score on interacting pairs is different (e.g. have a larger variance) than that of the non-interacting ones. 

See Additional file 4 for source code and data sets needed to replicate the experiments.

## Conclusions

We found that for a set of simulated and false interaction-imputed experimental data sets, the utility of replication groups as described by Gayan et al. (2008) does not improve upon the direct scoring of SNP interactions. In all our experiments, estimating interaction scores directly from the entire data set performed consistently better.

The aim of using replication groups was to decrease the number of false positive cases at expense of lower power of the test. As we have shown, the same decrease, but with a higher power, can be achieved with the standard method by simply raising the significance threshold. Alternatively, at the same power of the test, the standard method will provide less false positives than the replication group method.

We would like to stress that our study investigated a particular data sampling approach used in HFCC. We show that either with the originally proposed scoring method or another SNP interaction measure, the utility of replication groups should be replaced by more effective direct estimation of interaction scores from the entire data set.

## Authors' contributions

MT, TC, JD, and BZ conceived the study and designed the experiments. MT performed the experiments. MT and BZ wrote the manuscript. All authors revised the manuscript and approved its final version.

## Supplementary Material

Additional file 1 **Performance graphs for all data sets**. Graphs presenting the dependency of false positive counts given the number of selected best candidate interactions for all 12 simulated and 5 GEO data sets. Click here for file

Additional file 2 **Performance graphs obtained with bootstrap sampling**. Graphs presenting the dependency of false positive counts given the number of selected best candidate interactions for all 12 simulated and 5 GEO data sets. In addition to direct scoring and scoring with replication groups we report results obtained with bootstrap sampling. Click here for file

Additional file 3 **Performance graphs for differently sized subsets of GSE6754**. Performance graphs for data subsets of 100, 200, 500, 1000, 2000, and 5000 samples drawn from GSE6754. Click here for file

Additional file 4 **Source code and data sets**. Source code and data sets needed to replicate the experiments. Click here for file
