# Supplementary material for: Does replication groups scoring reduce false positive rate in SNP interaction discovery?
Source: BMC Genomics. 2010 Jan 22;11:58. doi: 10.1186/1471-2164-11-58 (PMC2823693; doi:10.1186/1471-2164-11-58)
Supplement: Additional file 1 — Performance graphs for all data sets. Graphs presenting the dependency of false positive counts given the number of selected best candidate interactions for all 12 simulated and 5 GEO data sets. [file 1471-2164-11-58-S1.ZIP › results.html]

Supplement to: Does replication groups scoring reduce false positive rate in
SNP interaction discovery?


## Results for all data sets

Graphs present the dependency of false positive counts
given the number of selected best candidate interactions.
A direct
scoring (solid curves) was compared to scoring with two (dashed
curves) or three (dotted curves) replication groups. Curves closer
to lower-right corner of the graph indicate better performance. The
axes are in logarithmic scale to emphasize the results for smaller
numbers of best candidates. The theoretically best and worst
possible performance curves are shown in light gray.

We provide results for 12 synthetic data sets and 5 experimental data sets.

### Synthetic data

We closely followed the data synthesis proposed by Ritchie et al. (2003).
The synthetic data sets were generated according to six two-SNP epistasis model. Unlike Ritchie et al. (2003), our data sets included multiple interactions, but such that each SNP was involved in interaction with at most one other SNP. Two different types of data sets with respect to the number of SNPs were crafted, each comprised 200 control and 200 disease cases:

1. 100 SNP data sets (**syn1**) with 24 interactions (four times all six
   epistasis models),- 500 SNP data sets (**syn2**) with 60 interactions (ten times all six
     epistasis models).

**syn1 (100 SNP data set) without noise**


**syn1 (100 SNP data set) with missing data noise**


**syn1 (100 SNP data set) with genotyping noise**


**syn1 (100 SNP data set) with phenocopies noise**


**syn1 (100 SNP data set) with genetic heterogeneity noise**


**syn1 (100 SNP data set) with all types of noise applied simultaneously**


**syn2 (500 SNP data set) without noise**


**syn2 (500 SNP data set) with missing data noise**


**syn2 (500 SNP data set) with genotyping noise**


**syn2 (500 SNP data set) with phenocopies noise**


**syn2 (500 SNP data set) with genetic heterogeneity noise**


**syn2 (500 SNP data set) with all types of noise applied simultaneously**


### Experimental data from Gene Expression Omnibus

**GSE6754**

Families with two individuals affected by autism spectrum disorders. Cases were classified to affected or unaffected. Due to HFCC software constraints, only the first 2,000 SNPs were considered and a stratified sample of 500 cases (292 affected, 208 unaffected) was used.


**GSE8054**

901 SNPs for each of the 121 cancerous samples and 87 controls.


**GSE8055**

1,189 SNPs for each of the 141 cancerous samples and 89 controls.


**GSE7226-GPL2004**

Platform designation GPL2004,
comprising 102 samples from mentally retarded children and 213
controls from their unaffected siblings or parents. The first
2,000 SNPs were considered.


**GSE7226-GPL2005**

Platform designation GPL2005,
comprising 103 samples from mentally retarded children and 210
controls from their unaffected siblings or parents. The first 2,000
SNPs were considered.
