# Supplementary material for: Does replication groups scoring reduce false positive rate in SNP interaction discovery?
Source: BMC Genomics. 2010 Jan 22;11:58. doi: 10.1186/1471-2164-11-58 (PMC2823693; doi:10.1186/1471-2164-11-58)
Supplement: Additional file 1 — Performance graphs for all data sets. Graphs presenting the dependency of false positive counts given the number of selected best candidate interactions for all 12 simulated and 5 GEO data sets. [file 1471-2164-11-58-S1.ZIP › links.htm]

About the supplement  
Results for all data sets  
 
