# Supplementary material for: Does replication groups scoring reduce false positive rate in SNP interaction discovery?
Source: BMC Genomics. 2010 Jan 22;11:58. doi: 10.1186/1471-2164-11-58 (PMC2823693; doi:10.1186/1471-2164-11-58)
Supplement: Additional file 1 — Performance graphs for all data sets. Graphs presenting the dependency of false positive counts given the number of selected best candidate interactions for all 12 simulated and 5 GEO data sets. [file 1471-2164-11-58-S1.ZIP › index.html]

Supplement to: Does replication groups scoring reduce false positive rate in
SNP interaction discovery?


## Supplement to: Does replication groups scoring reduce false positive rate in SNP interaction discovery?

### Results for all data sets

In the article we show results for the three data sets only. Here we provide graphs presenting the dependency of false positive counts
given the number of selected best candidate interactions for all data set.

### Source code and data sets

We also provide source code and the data sets needed
to perform the experiments. A modern 32-bit modern Linux system is required
to run the experiments. Instructions for installation and are in 
README.txt file.

Download source code with the data sets.
