# Supplementary figures and images for: Does replication groups scoring reduce false positive rate in SNP interaction discovery?
Source: BMC Genomics. 2010 Jan 22;11:58. doi: 10.1186/1471-2164-11-58 (PMC2823693; doi:10.1186/1471-2164-11-58)

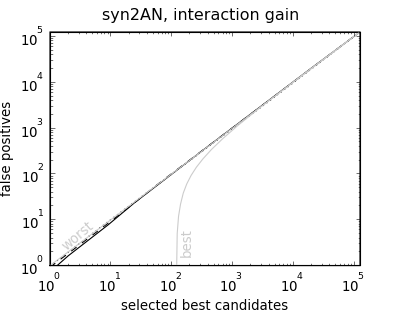

Supplement: Additional file 1 — Performance graphs for all data sets. Graphs presenting the dependency of false positive counts given the number of selected best candidate interactions for all 12 simulated and 5 GEO data sets. [file 1471-2164-11-58-S1.ZIP › pngres/fpgraph_model2AN_int.png]

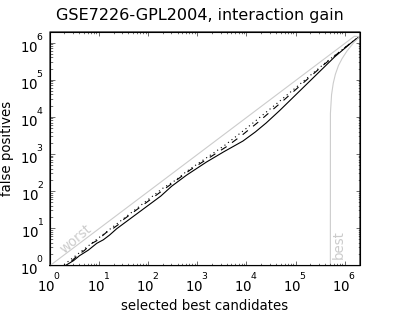

Supplement: Additional file 1 — Performance graphs for all data sets. Graphs presenting the dependency of false positive counts given the number of selected best candidate interactions for all 12 simulated and 5 GEO data sets. [file 1471-2164-11-58-S1.ZIP › pngres/fpgraph_gse7226-gpl2004_2000at.tab_int.png]

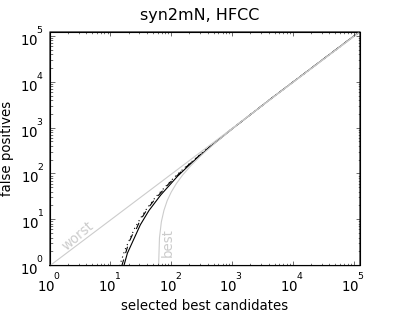

Supplement: Additional file 1 — Performance graphs for all data sets. Graphs presenting the dependency of false positive counts given the number of selected best candidate interactions for all 12 simulated and 5 GEO data sets. [file 1471-2164-11-58-S1.ZIP › pngres/fpgraph_model2mN_hfcc.png]

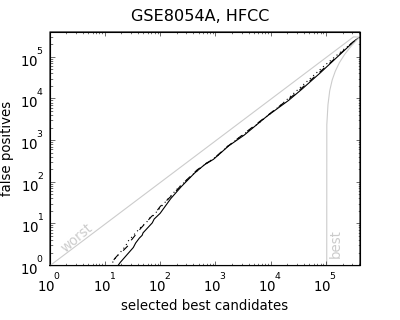

Supplement: Additional file 1 — Performance graphs for all data sets. Graphs presenting the dependency of false positive counts given the number of selected best candidate interactions for all 12 simulated and 5 GEO data sets. [file 1471-2164-11-58-S1.ZIP › pngres/fpgraph_gse8054a.tab_hfcc.png]

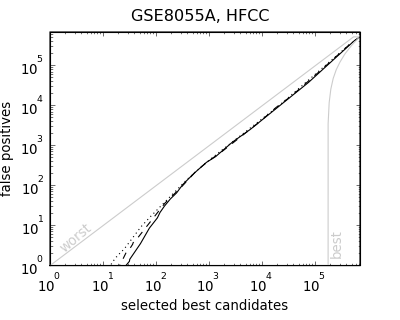

Supplement: Additional file 1 — Performance graphs for all data sets. Graphs presenting the dependency of false positive counts given the number of selected best candidate interactions for all 12 simulated and 5 GEO data sets. [file 1471-2164-11-58-S1.ZIP › pngres/fpgraph_gse8055a.tab_hfcc.png]

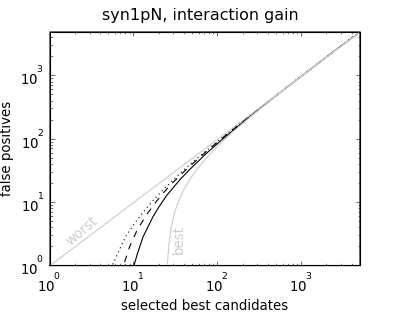

Supplement: Additional file 1 — Performance graphs for all data sets. Graphs presenting the dependency of false positive counts given the number of selected best candidate interactions for all 12 simulated and 5 GEO data sets. [file 1471-2164-11-58-S1.ZIP › pngres/fpgraph_model1pN_int.png]

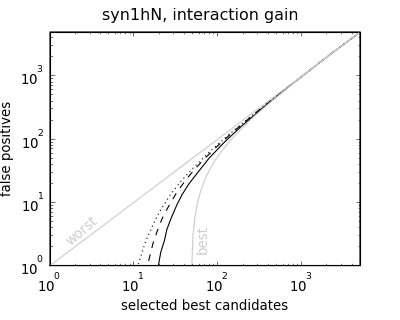

Supplement: Additional file 1 — Performance graphs for all data sets. Graphs presenting the dependency of false positive counts given the number of selected best candidate interactions for all 12 simulated and 5 GEO data sets. [file 1471-2164-11-58-S1.ZIP › pngres/fpgraph_model1hN_int.png]

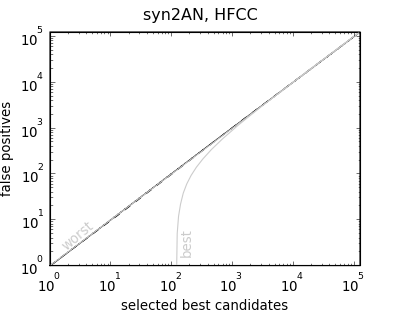

Supplement: Additional file 1 — Performance graphs for all data sets. Graphs presenting the dependency of false positive counts given the number of selected best candidate interactions for all 12 simulated and 5 GEO data sets. [file 1471-2164-11-58-S1.ZIP › pngres/fpgraph_model2AN_hfcc.png]

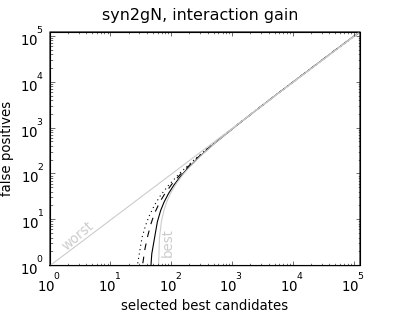

Supplement: Additional file 1 — Performance graphs for all data sets. Graphs presenting the dependency of false positive counts given the number of selected best candidate interactions for all 12 simulated and 5 GEO data sets. [file 1471-2164-11-58-S1.ZIP › pngres/fpgraph_model2gN_int.png]

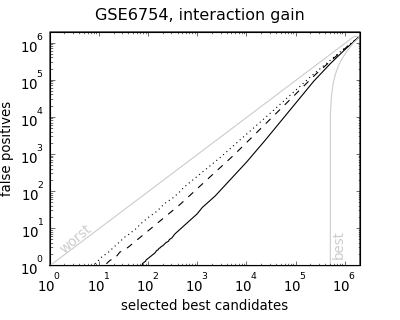

Supplement: Additional file 1 — Performance graphs for all data sets. Graphs presenting the dependency of false positive counts given the number of selected best candidate interactions for all 12 simulated and 5 GEO data sets. [file 1471-2164-11-58-S1.ZIP › pngres/fpgraph_gse6754_status_500ex_2000at.tab_int.png]

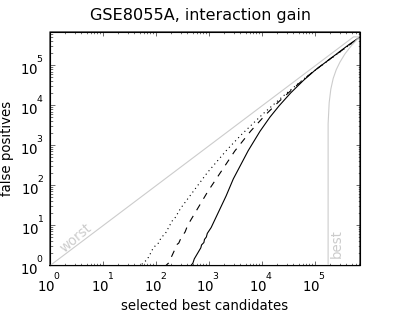

Supplement: Additional file 1 — Performance graphs for all data sets. Graphs presenting the dependency of false positive counts given the number of selected best candidate interactions for all 12 simulated and 5 GEO data sets. [file 1471-2164-11-58-S1.ZIP › pngres/fpgraph_gse8055a.tab_int.png]

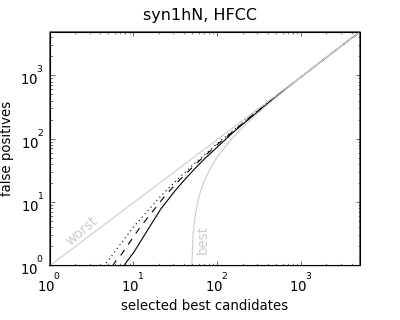

Supplement: Additional file 1 — Performance graphs for all data sets. Graphs presenting the dependency of false positive counts given the number of selected best candidate interactions for all 12 simulated and 5 GEO data sets. [file 1471-2164-11-58-S1.ZIP › pngres/fpgraph_model1hN_hfcc.png]

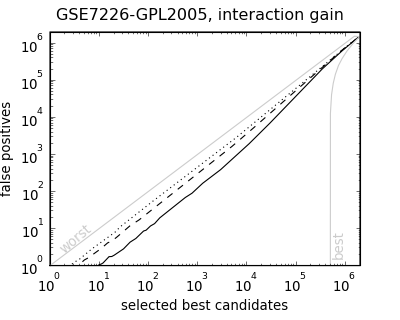

Supplement: Additional file 1 — Performance graphs for all data sets. Graphs presenting the dependency of false positive counts given the number of selected best candidate interactions for all 12 simulated and 5 GEO data sets. [file 1471-2164-11-58-S1.ZIP › pngres/fpgraph_gse7226-gpl2005_2000at.tab_int.png]

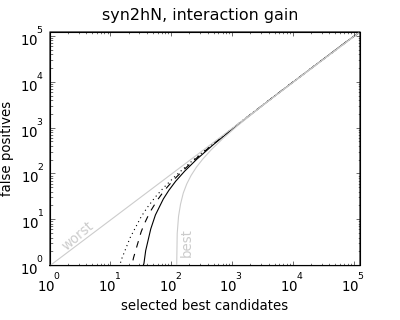

Supplement: Additional file 1 — Performance graphs for all data sets. Graphs presenting the dependency of false positive counts given the number of selected best candidate interactions for all 12 simulated and 5 GEO data sets. [file 1471-2164-11-58-S1.ZIP › pngres/fpgraph_model2hN_int.png]

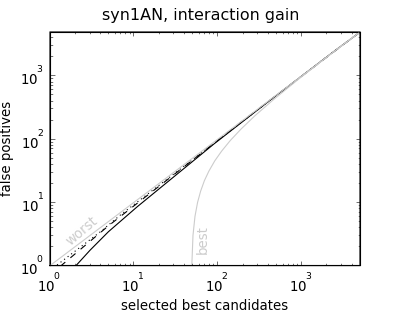

Supplement: Additional file 1 — Performance graphs for all data sets. Graphs presenting the dependency of false positive counts given the number of selected best candidate interactions for all 12 simulated and 5 GEO data sets. [file 1471-2164-11-58-S1.ZIP › pngres/fpgraph_model1AN_int.png]

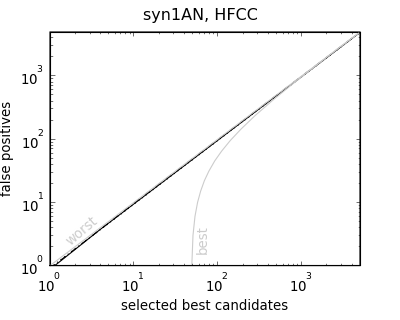

Supplement: Additional file 1 — Performance graphs for all data sets. Graphs presenting the dependency of false positive counts given the number of selected best candidate interactions for all 12 simulated and 5 GEO data sets. [file 1471-2164-11-58-S1.ZIP › pngres/fpgraph_model1AN_hfcc.png]

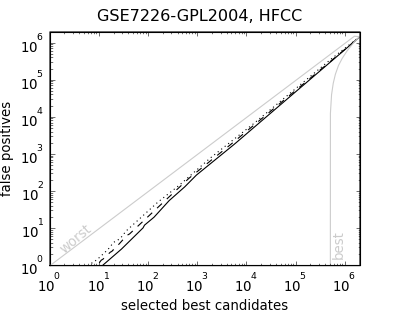

Supplement: Additional file 1 — Performance graphs for all data sets. Graphs presenting the dependency of false positive counts given the number of selected best candidate interactions for all 12 simulated and 5 GEO data sets. [file 1471-2164-11-58-S1.ZIP › pngres/fpgraph_gse7226-gpl2004_2000at.tab_hfcc.png]

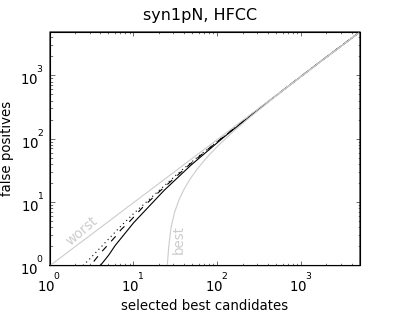

Supplement: Additional file 1 — Performance graphs for all data sets. Graphs presenting the dependency of false positive counts given the number of selected best candidate interactions for all 12 simulated and 5 GEO data sets. [file 1471-2164-11-58-S1.ZIP › pngres/fpgraph_model1pN_hfcc.png]

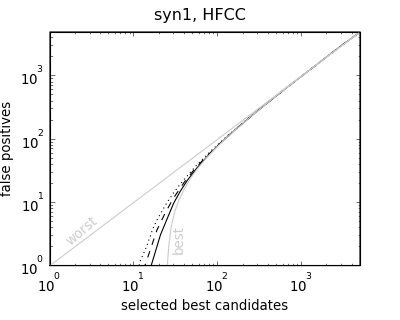

Supplement: Additional file 1 — Performance graphs for all data sets. Graphs presenting the dependency of false positive counts given the number of selected best candidate interactions for all 12 simulated and 5 GEO data sets. [file 1471-2164-11-58-S1.ZIP › pngres/fpgraph_model1WN_hfcc.png]

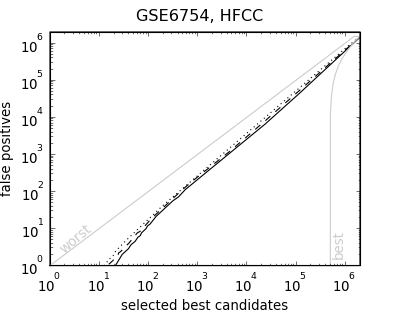

Supplement: Additional file 1 — Performance graphs for all data sets. Graphs presenting the dependency of false positive counts given the number of selected best candidate interactions for all 12 simulated and 5 GEO data sets. [file 1471-2164-11-58-S1.ZIP › pngres/fpgraph_gse6754_status_500ex_2000at.tab_hfcc.png]

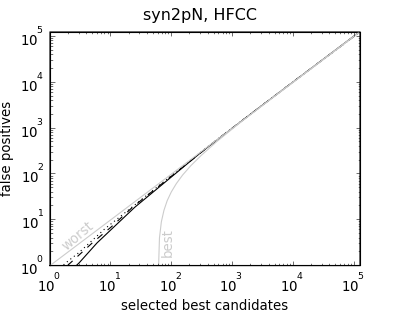

Supplement: Additional file 1 — Performance graphs for all data sets. Graphs presenting the dependency of false positive counts given the number of selected best candidate interactions for all 12 simulated and 5 GEO data sets. [file 1471-2164-11-58-S1.ZIP › pngres/fpgraph_model2pN_hfcc.png]

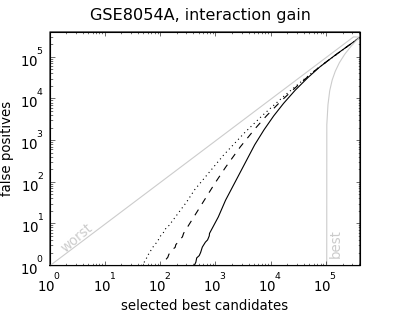

Supplement: Additional file 1 — Performance graphs for all data sets. Graphs presenting the dependency of false positive counts given the number of selected best candidate interactions for all 12 simulated and 5 GEO data sets. [file 1471-2164-11-58-S1.ZIP › pngres/fpgraph_gse8054a.tab_int.png]

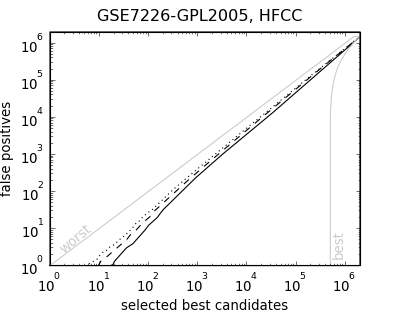

Supplement: Additional file 1 — Performance graphs for all data sets. Graphs presenting the dependency of false positive counts given the number of selected best candidate interactions for all 12 simulated and 5 GEO data sets. [file 1471-2164-11-58-S1.ZIP › pngres/fpgraph_gse7226-gpl2005_2000at.tab_hfcc.png]

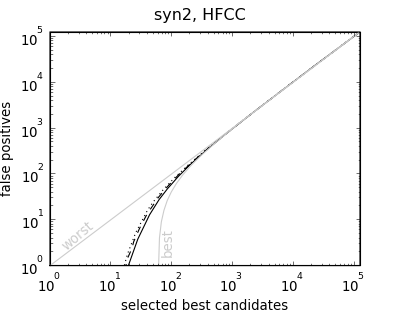

Supplement: Additional file 1 — Performance graphs for all data sets. Graphs presenting the dependency of false positive counts given the number of selected best candidate interactions for all 12 simulated and 5 GEO data sets. [file 1471-2164-11-58-S1.ZIP › pngres/fpgraph_model2WN_hfcc.png]

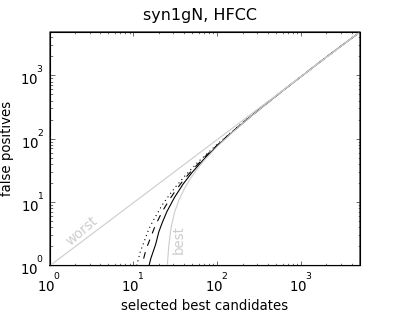

Supplement: Additional file 1 — Performance graphs for all data sets. Graphs presenting the dependency of false positive counts given the number of selected best candidate interactions for all 12 simulated and 5 GEO data sets. [file 1471-2164-11-58-S1.ZIP › pngres/fpgraph_model1gN_hfcc.png]

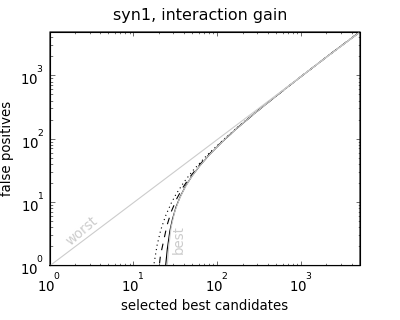

Supplement: Additional file 1 — Performance graphs for all data sets. Graphs presenting the dependency of false positive counts given the number of selected best candidate interactions for all 12 simulated and 5 GEO data sets. [file 1471-2164-11-58-S1.ZIP › pngres/fpgraph_model1WN_int.png]

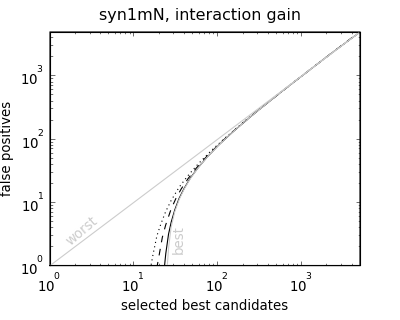

Supplement: Additional file 1 — Performance graphs for all data sets. Graphs presenting the dependency of false positive counts given the number of selected best candidate interactions for all 12 simulated and 5 GEO data sets. [file 1471-2164-11-58-S1.ZIP › pngres/fpgraph_model1mN_int.png]

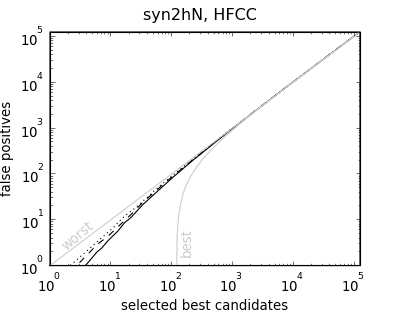

Supplement: Additional file 1 — Performance graphs for all data sets. Graphs presenting the dependency of false positive counts given the number of selected best candidate interactions for all 12 simulated and 5 GEO data sets. [file 1471-2164-11-58-S1.ZIP › pngres/fpgraph_model2hN_hfcc.png]

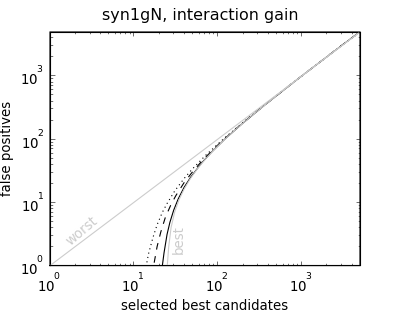

Supplement: Additional file 1 — Performance graphs for all data sets. Graphs presenting the dependency of false positive counts given the number of selected best candidate interactions for all 12 simulated and 5 GEO data sets. [file 1471-2164-11-58-S1.ZIP › pngres/fpgraph_model1gN_int.png]

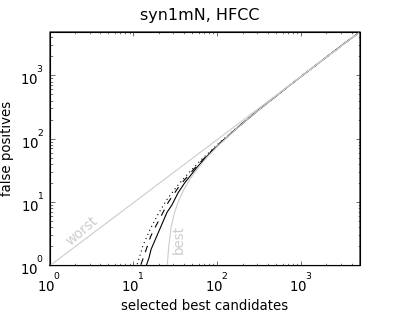

Supplement: Additional file 1 — Performance graphs for all data sets. Graphs presenting the dependency of false positive counts given the number of selected best candidate interactions for all 12 simulated and 5 GEO data sets. [file 1471-2164-11-58-S1.ZIP › pngres/fpgraph_model1mN_hfcc.png]

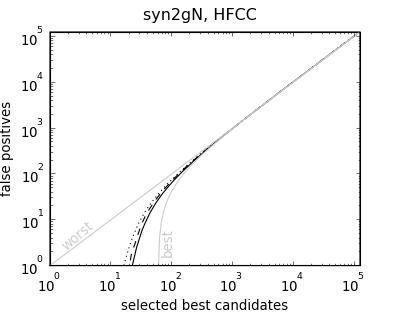

Supplement: Additional file 1 — Performance graphs for all data sets. Graphs presenting the dependency of false positive counts given the number of selected best candidate interactions for all 12 simulated and 5 GEO data sets. [file 1471-2164-11-58-S1.ZIP › pngres/fpgraph_model2gN_hfcc.png]

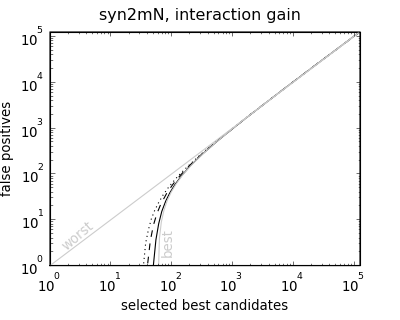

Supplement: Additional file 1 — Performance graphs for all data sets. Graphs presenting the dependency of false positive counts given the number of selected best candidate interactions for all 12 simulated and 5 GEO data sets. [file 1471-2164-11-58-S1.ZIP › pngres/fpgraph_model2mN_int.png]

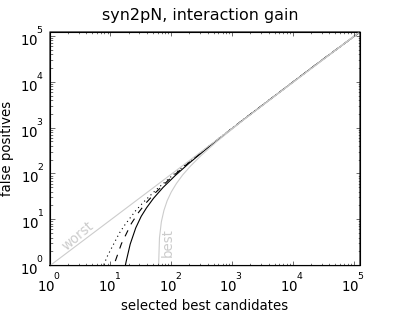

Supplement: Additional file 1 — Performance graphs for all data sets. Graphs presenting the dependency of false positive counts given the number of selected best candidate interactions for all 12 simulated and 5 GEO data sets. [file 1471-2164-11-58-S1.ZIP › pngres/fpgraph_model2pN_int.png]

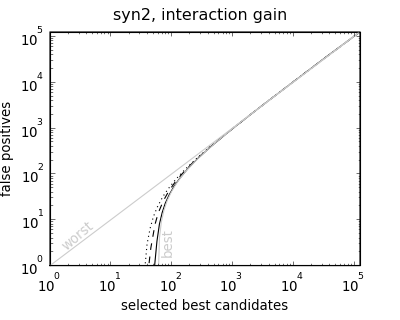

Supplement: Additional file 1 — Performance graphs for all data sets. Graphs presenting the dependency of false positive counts given the number of selected best candidate interactions for all 12 simulated and 5 GEO data sets. [file 1471-2164-11-58-S1.ZIP › pngres/fpgraph_model2WN_int.png]

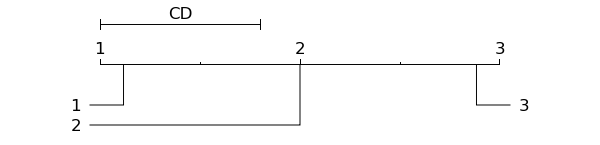

Supplement: Additional file 1 — Performance graphs for all data sets. Graphs presenting the dependency of false positive counts given the number of selected best candidate interactions for all 12 simulated and 5 GEO data sets. [file 1471-2164-11-58-S1.ZIP › res/interaction gain.png]

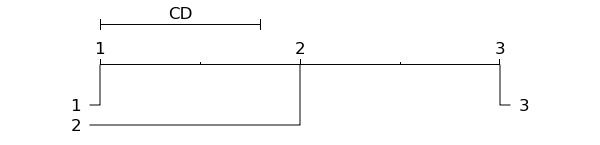

Supplement: Additional file 1 — Performance graphs for all data sets. Graphs presenting the dependency of false positive counts given the number of selected best candidate interactions for all 12 simulated and 5 GEO data sets. [file 1471-2164-11-58-S1.ZIP › res/HFCC.png]

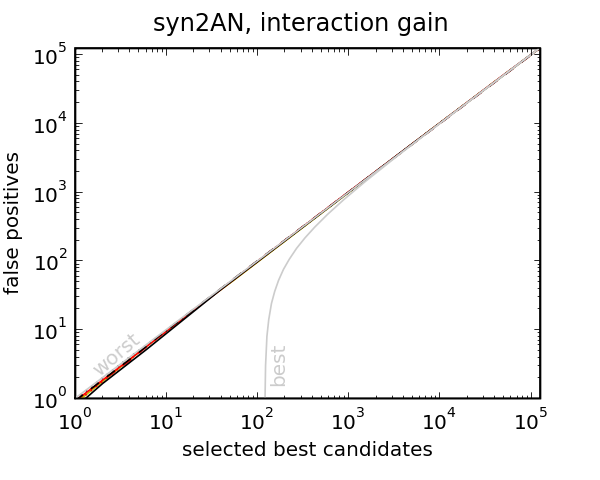

Supplement: Additional file 2 — Performance graphs obtained with bootstrap sampling. Graphs presenting the dependency of false positive counts given the number of selected best candidate interactions for all 12 simulated and 5 GEO data sets. In addition to direct scoring and scoring with replication groups we report results obtained with bootstrap sampling. [file 1471-2164-11-58-S2.ZIP › pngres_bootstrap/fpgraph_model2AN_int.png]

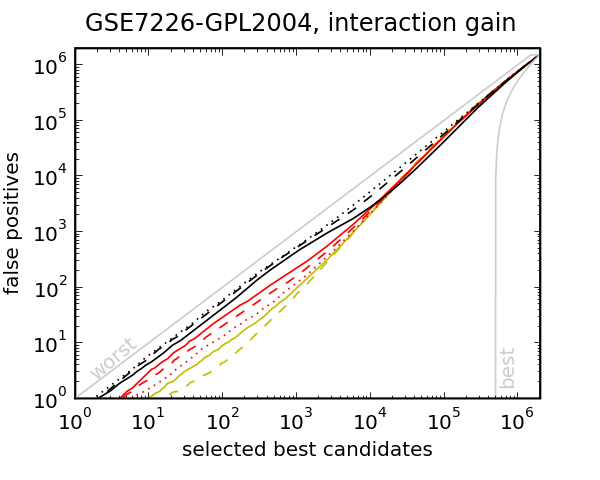

Supplement: Additional file 2 — Performance graphs obtained with bootstrap sampling. Graphs presenting the dependency of false positive counts given the number of selected best candidate interactions for all 12 simulated and 5 GEO data sets. In addition to direct scoring and scoring with replication groups we report results obtained with bootstrap sampling. [file 1471-2164-11-58-S2.ZIP › pngres_bootstrap/fpgraph_gse7226-gpl2004_2000at.tab_int.png]

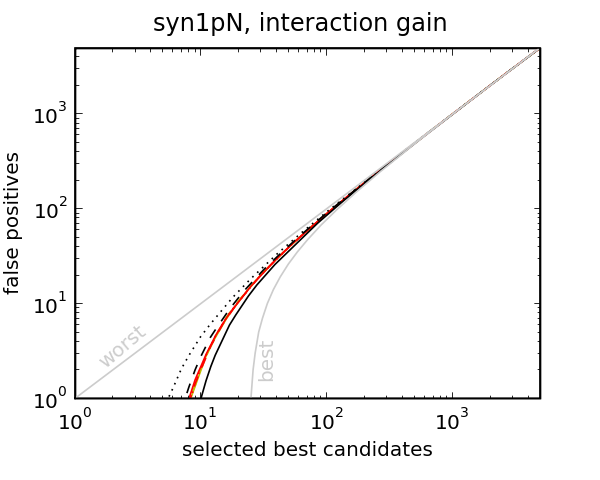

Supplement: Additional file 2 — Performance graphs obtained with bootstrap sampling. Graphs presenting the dependency of false positive counts given the number of selected best candidate interactions for all 12 simulated and 5 GEO data sets. In addition to direct scoring and scoring with replication groups we report results obtained with bootstrap sampling. [file 1471-2164-11-58-S2.ZIP › pngres_bootstrap/fpgraph_model1pN_int.png]

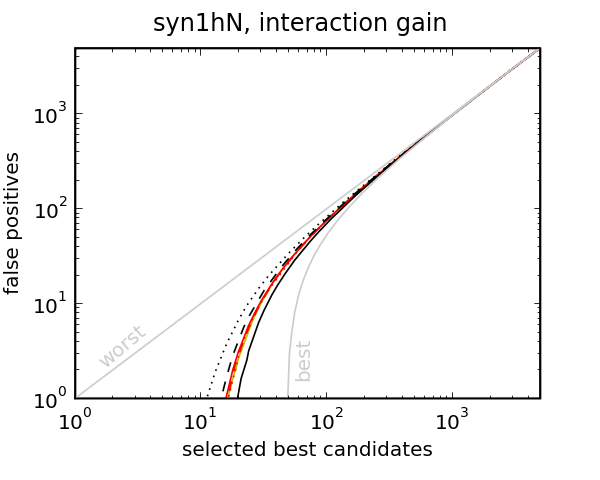

Supplement: Additional file 2 — Performance graphs obtained with bootstrap sampling. Graphs presenting the dependency of false positive counts given the number of selected best candidate interactions for all 12 simulated and 5 GEO data sets. In addition to direct scoring and scoring with replication groups we report results obtained with bootstrap sampling. [file 1471-2164-11-58-S2.ZIP › pngres_bootstrap/fpgraph_model1hN_int.png]

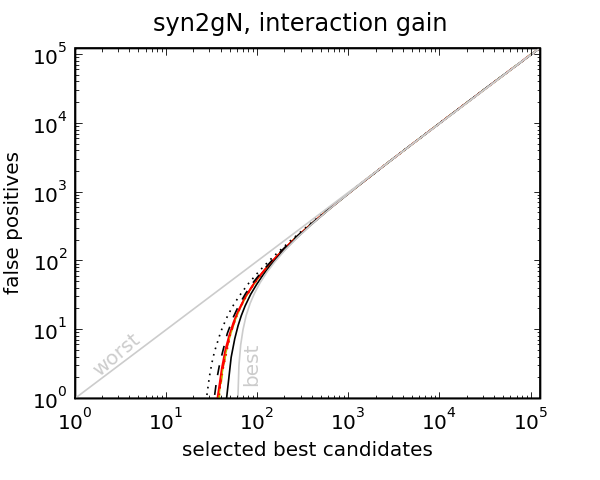

Supplement: Additional file 2 — Performance graphs obtained with bootstrap sampling. Graphs presenting the dependency of false positive counts given the number of selected best candidate interactions for all 12 simulated and 5 GEO data sets. In addition to direct scoring and scoring with replication groups we report results obtained with bootstrap sampling. [file 1471-2164-11-58-S2.ZIP › pngres_bootstrap/fpgraph_model2gN_int.png]

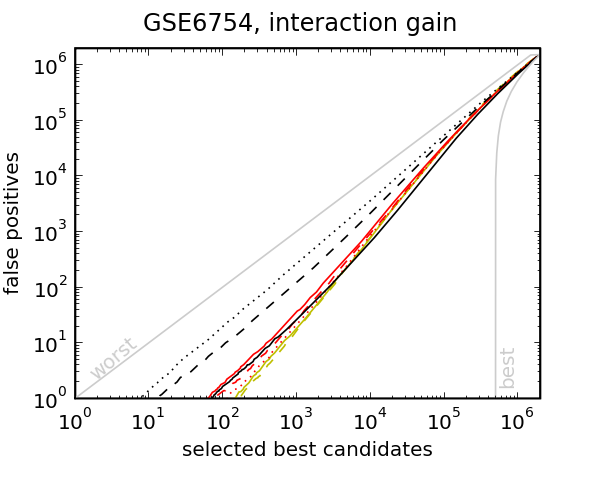

Supplement: Additional file 2 — Performance graphs obtained with bootstrap sampling. Graphs presenting the dependency of false positive counts given the number of selected best candidate interactions for all 12 simulated and 5 GEO data sets. In addition to direct scoring and scoring with replication groups we report results obtained with bootstrap sampling. [file 1471-2164-11-58-S2.ZIP › pngres_bootstrap/fpgraph_gse6754_status_500ex_2000at.tab_int.png]

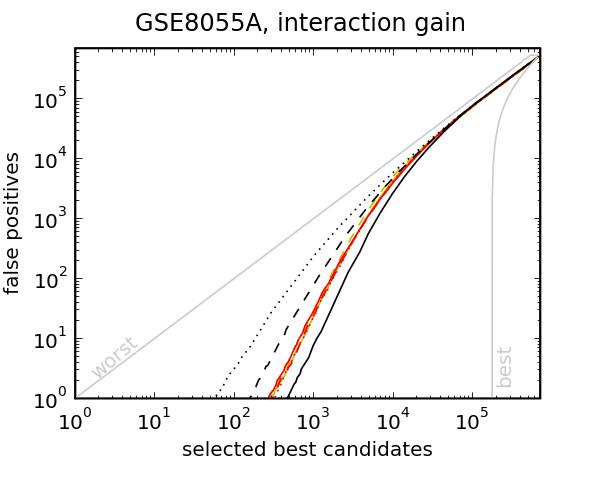

Supplement: Additional file 2 — Performance graphs obtained with bootstrap sampling. Graphs presenting the dependency of false positive counts given the number of selected best candidate interactions for all 12 simulated and 5 GEO data sets. In addition to direct scoring and scoring with replication groups we report results obtained with bootstrap sampling. [file 1471-2164-11-58-S2.ZIP › pngres_bootstrap/fpgraph_gse8055a.tab_int.png]

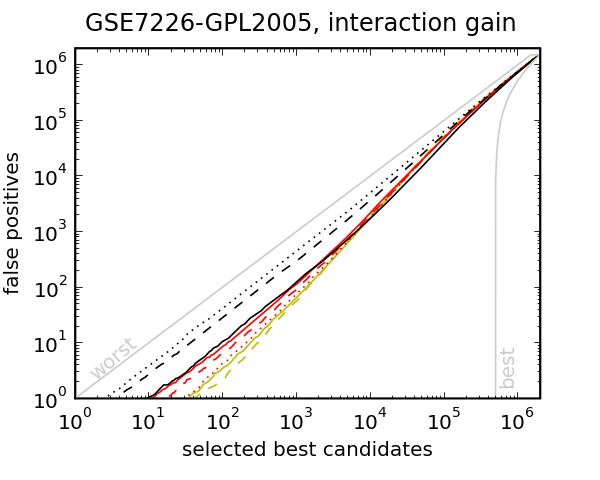

Supplement: Additional file 2 — Performance graphs obtained with bootstrap sampling. Graphs presenting the dependency of false positive counts given the number of selected best candidate interactions for all 12 simulated and 5 GEO data sets. In addition to direct scoring and scoring with replication groups we report results obtained with bootstrap sampling. [file 1471-2164-11-58-S2.ZIP › pngres_bootstrap/fpgraph_gse7226-gpl2005_2000at.tab_int.png]

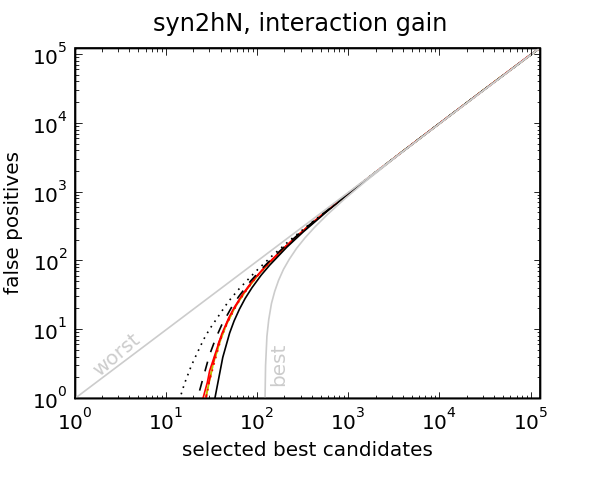

Supplement: Additional file 2 — Performance graphs obtained with bootstrap sampling. Graphs presenting the dependency of false positive counts given the number of selected best candidate interactions for all 12 simulated and 5 GEO data sets. In addition to direct scoring and scoring with replication groups we report results obtained with bootstrap sampling. [file 1471-2164-11-58-S2.ZIP › pngres_bootstrap/fpgraph_model2hN_int.png]

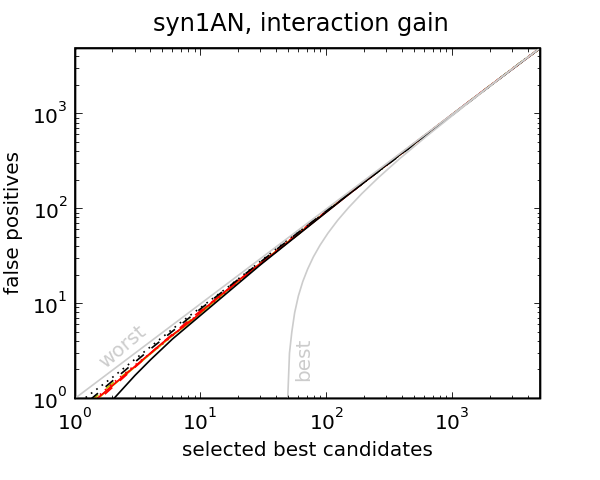

Supplement: Additional file 2 — Performance graphs obtained with bootstrap sampling. Graphs presenting the dependency of false positive counts given the number of selected best candidate interactions for all 12 simulated and 5 GEO data sets. In addition to direct scoring and scoring with replication groups we report results obtained with bootstrap sampling. [file 1471-2164-11-58-S2.ZIP › pngres_bootstrap/fpgraph_model1AN_int.png]

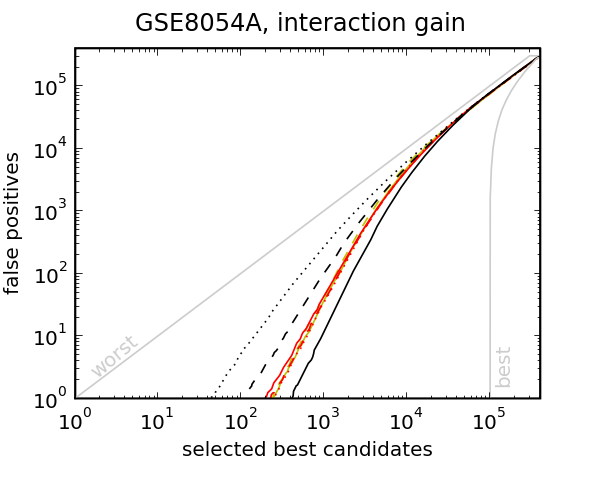

Supplement: Additional file 2 — Performance graphs obtained with bootstrap sampling. Graphs presenting the dependency of false positive counts given the number of selected best candidate interactions for all 12 simulated and 5 GEO data sets. In addition to direct scoring and scoring with replication groups we report results obtained with bootstrap sampling. [file 1471-2164-11-58-S2.ZIP › pngres_bootstrap/fpgraph_gse8054a.tab_int.png]

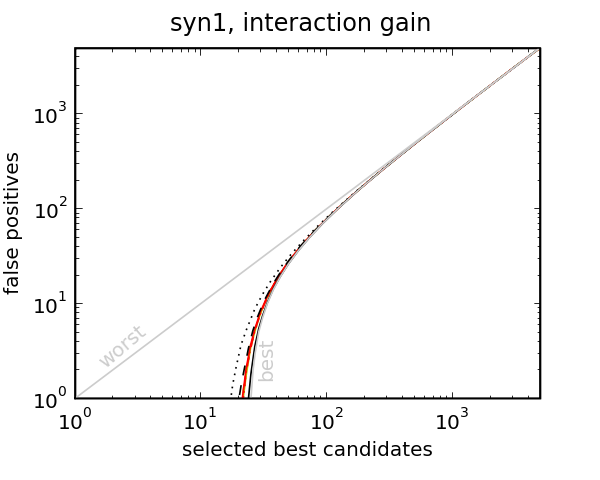

Supplement: Additional file 2 — Performance graphs obtained with bootstrap sampling. Graphs presenting the dependency of false positive counts given the number of selected best candidate interactions for all 12 simulated and 5 GEO data sets. In addition to direct scoring and scoring with replication groups we report results obtained with bootstrap sampling. [file 1471-2164-11-58-S2.ZIP › pngres_bootstrap/fpgraph_model1WN_int.png]

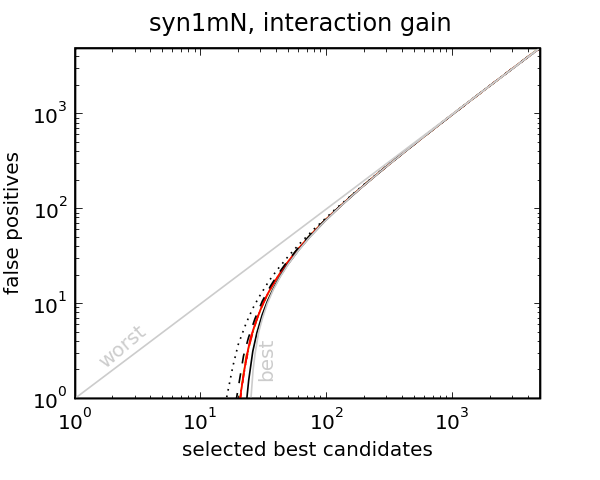

Supplement: Additional file 2 — Performance graphs obtained with bootstrap sampling. Graphs presenting the dependency of false positive counts given the number of selected best candidate interactions for all 12 simulated and 5 GEO data sets. In addition to direct scoring and scoring with replication groups we report results obtained with bootstrap sampling. [file 1471-2164-11-58-S2.ZIP › pngres_bootstrap/fpgraph_model1mN_int.png]

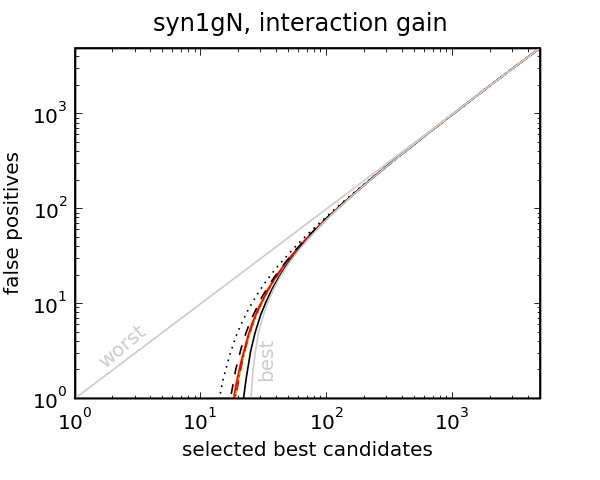

Supplement: Additional file 2 — Performance graphs obtained with bootstrap sampling. Graphs presenting the dependency of false positive counts given the number of selected best candidate interactions for all 12 simulated and 5 GEO data sets. In addition to direct scoring and scoring with replication groups we report results obtained with bootstrap sampling. [file 1471-2164-11-58-S2.ZIP › pngres_bootstrap/fpgraph_model1gN_int.png]

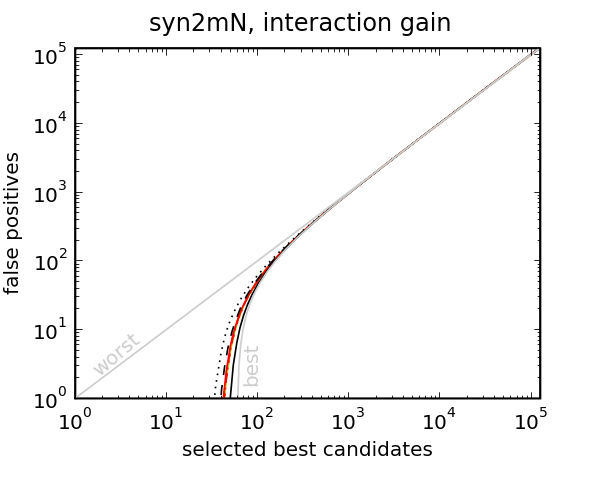

Supplement: Additional file 2 — Performance graphs obtained with bootstrap sampling. Graphs presenting the dependency of false positive counts given the number of selected best candidate interactions for all 12 simulated and 5 GEO data sets. In addition to direct scoring and scoring with replication groups we report results obtained with bootstrap sampling. [file 1471-2164-11-58-S2.ZIP › pngres_bootstrap/fpgraph_model2mN_int.png]

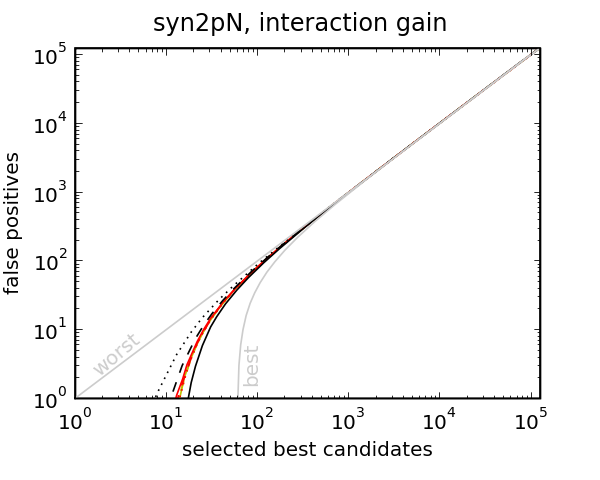

Supplement: Additional file 2 — Performance graphs obtained with bootstrap sampling. Graphs presenting the dependency of false positive counts given the number of selected best candidate interactions for all 12 simulated and 5 GEO data sets. In addition to direct scoring and scoring with replication groups we report results obtained with bootstrap sampling. [file 1471-2164-11-58-S2.ZIP › pngres_bootstrap/fpgraph_model2pN_int.png]

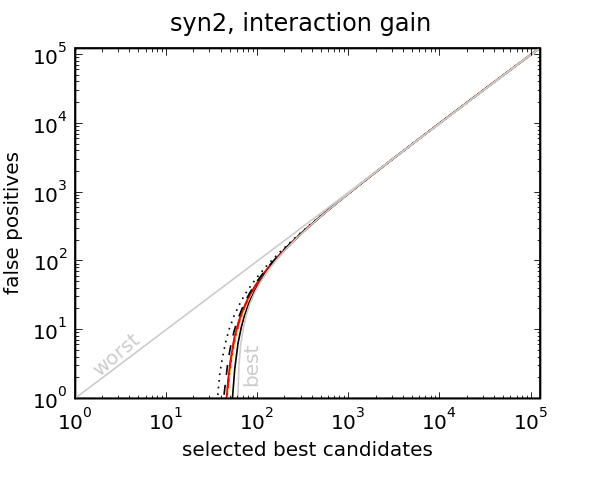

Supplement: Additional file 2 — Performance graphs obtained with bootstrap sampling. Graphs presenting the dependency of false positive counts given the number of selected best candidate interactions for all 12 simulated and 5 GEO data sets. In addition to direct scoring and scoring with replication groups we report results obtained with bootstrap sampling. [file 1471-2164-11-58-S2.ZIP › pngres_bootstrap/fpgraph_model2WN_int.png]

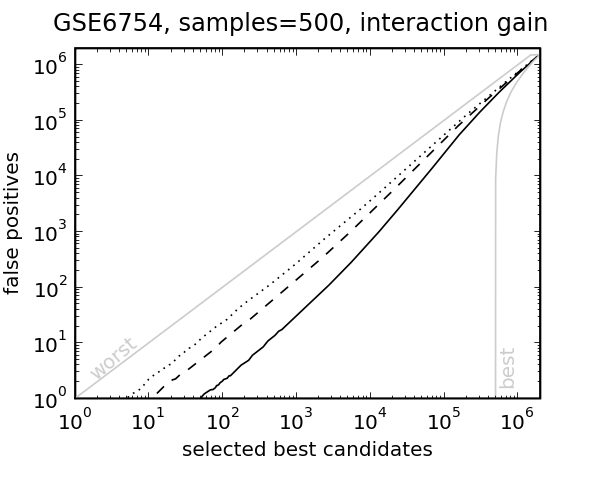

Supplement: Additional file 3 — Performance graphs for differently sized subsets of GSE6754. Performance graphs for data subsets of 100, 200, 500, 1000, 2000, and 5000 samples drawn from GSE6754. [file 1471-2164-11-58-S3.ZIP › pngres_big/fpgraph_gse6754_status_2000a.tab.sized500_int.png]

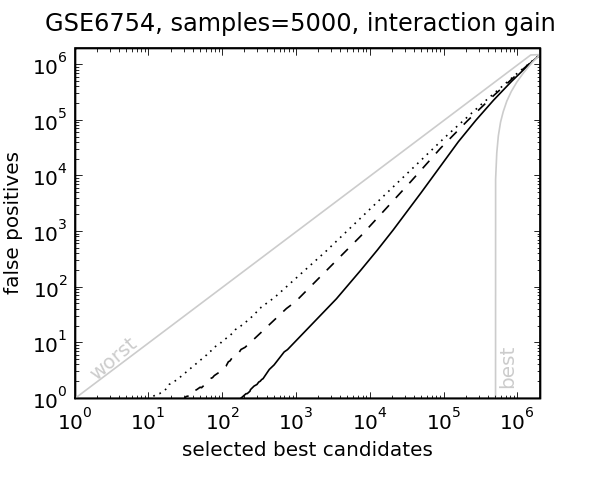

Supplement: Additional file 3 — Performance graphs for differently sized subsets of GSE6754. Performance graphs for data subsets of 100, 200, 500, 1000, 2000, and 5000 samples drawn from GSE6754. [file 1471-2164-11-58-S3.ZIP › pngres_big/fpgraph_gse6754_status_2000a.tab.sized5000_int.png]

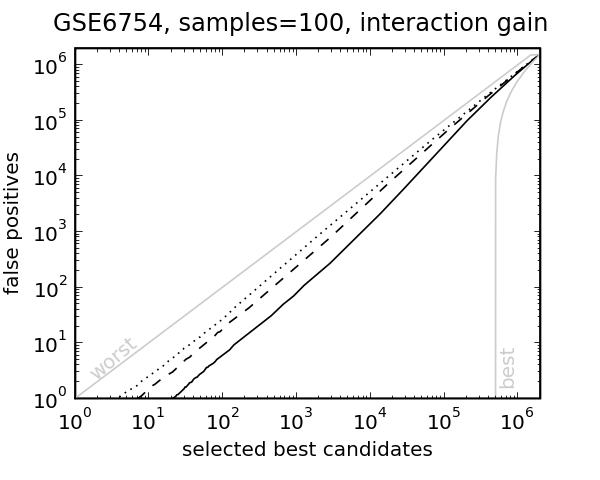

Supplement: Additional file 3 — Performance graphs for differently sized subsets of GSE6754. Performance graphs for data subsets of 100, 200, 500, 1000, 2000, and 5000 samples drawn from GSE6754. [file 1471-2164-11-58-S3.ZIP › pngres_big/fpgraph_gse6754_status_2000a.tab.sized100_int.png]

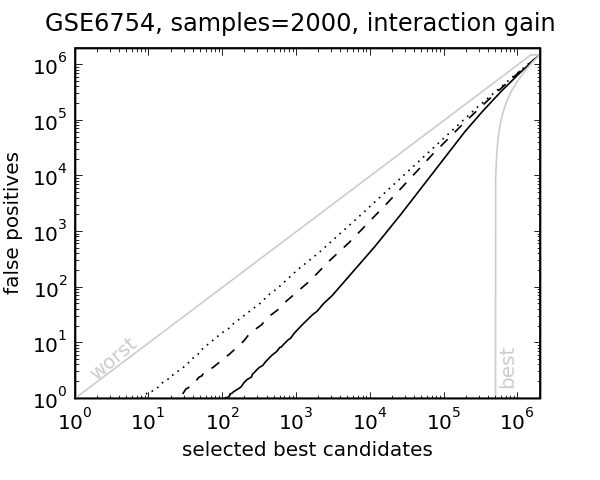

Supplement: Additional file 3 — Performance graphs for differently sized subsets of GSE6754. Performance graphs for data subsets of 100, 200, 500, 1000, 2000, and 5000 samples drawn from GSE6754. [file 1471-2164-11-58-S3.ZIP › pngres_big/fpgraph_gse6754_status_2000a.tab.sized2000_int.png]

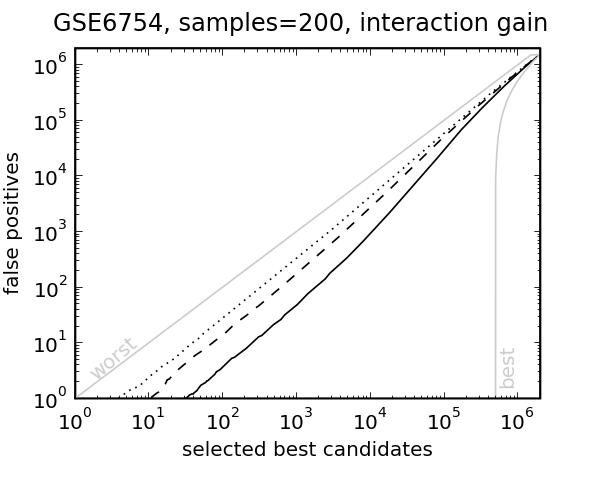

Supplement: Additional file 3 — Performance graphs for differently sized subsets of GSE6754. Performance graphs for data subsets of 100, 200, 500, 1000, 2000, and 5000 samples drawn from GSE6754. [file 1471-2164-11-58-S3.ZIP › pngres_big/fpgraph_gse6754_status_2000a.tab.sized200_int.png]

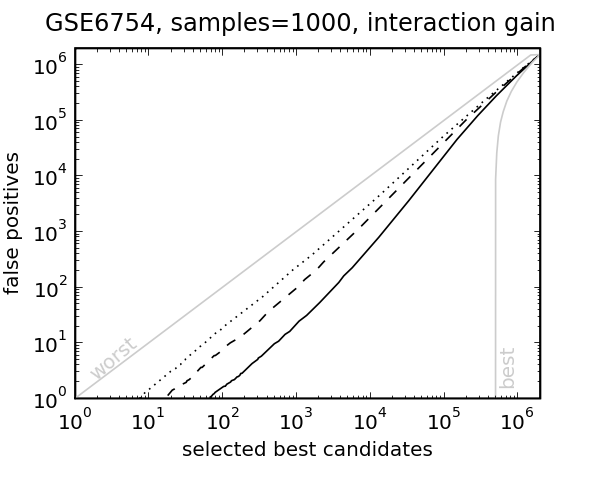

Supplement: Additional file 3 — Performance graphs for differently sized subsets of GSE6754. Performance graphs for data subsets of 100, 200, 500, 1000, 2000, and 5000 samples drawn from GSE6754. [file 1471-2164-11-58-S3.ZIP › pngres_big/fpgraph_gse6754_status_2000a.tab.sized1000_int.png]
