# Supplementary material for: Does replication groups scoring reduce false positive rate in SNP interaction discovery?
Source: BMC Genomics. 2010 Jan 22;11:58. doi: 10.1186/1471-2164-11-58 (PMC2823693; doi:10.1186/1471-2164-11-58)
Supplement: Additional file 2 — Performance graphs obtained with bootstrap sampling. Graphs presenting the dependency of false positive counts given the number of selected best candidate interactions for all 12 simulated and 5 GEO data sets. In addition to direct scoring and scoring with replication groups we report results obtained with bootstrap sampling. [file 1471-2164-11-58-S2.ZIP › results_bootstrap.html]

Supplement to: Does replication groups scoring reduce false positive rate in
SNP interaction discovery?


## Performance graphs obtained with bootstrap sampling

Graphs present the dependency of false positive counts
given the number of selected best candidate interactions.
Curves closer to lower-right corner of the graph indicate better performance. The
axes are in logarithmic scale to emphasize the results for smaller
numbers of best candidates.

We provide results for 12 simulated data sets and 5 experimental data sets.

### Curve legend

light gray - theoretically best and worst possible performance curves  
black solid - direct scoring  
black dashed - scoring with two replication groups  
black dotted - scoring with three replication groups  
red solid - scoring with 10 bootstrap samples instead of data partitioning  
red dashed - scoring with 20 bootstrap samples instead of data partitioning  
red dotted - scoring with 50 bootstrap samples instead of data partitioning  
yellow solid - scoring with 100 bootstrap samples instead of data partitioning  
yellow dashed - scoring with 500 bootstrap samples instead of data partitioning

### Simulated data

We followed the data synthesis by Ritchie et al. (2003).
The simulated data sets were generated according to six two-SNP epistasis model. Unlike Ritchie et al. (2003), our data sets included multiple interactions, but such that each SNP was involved in interaction with at most one other SNP. Two different types of data sets with respect to the number of SNPs were crafted, each comprised 200 control and 200 disease samples:

1. 100 SNP data sets (**syn1**) with 24 interactions (four times all six epistasis models),- 500 SNP data sets (**syn2**) with 60 interactions (ten times all six epistasis models).

**syn1 (100 SNP data set) without noise**

**syn1 (100 SNP data set) with missing data noise**

**syn1 (100 SNP data set) with genotyping noise**

**syn1 (100 SNP data set) with phenocopies noise**

**syn1 (100 SNP data set) with genetic heterogeneity noise**

**syn1 (100 SNP data set) with all types of noise applied simultaneously**

**syn2 (500 SNP data set) without noise**

**syn2 (500 SNP data set) with missing data noise**

**syn2 (500 SNP data set) with genotyping noise**

**syn2 (500 SNP data set) with phenocopies noise**

**syn2 (500 SNP data set) with genetic heterogeneity noise**

**syn2 (500 SNP data set) with all types of noise applied simultaneously**

### Experimental data from Gene Expression Omnibus

**GSE6754**

Families with two individuals affected by autism spectrum disorders. Individuals were classified to affected or unaffected. Due to HFCC software constraints, only the first 2,000 SNPs were considered and a stratified sample of 500 individuals (292 affected, 208 unaffected) was used.

**GSE8054**

901 SNPs for each of the 121 cancerous samples and 87 controls.

**GSE8055**

1,189 SNPs for each of the 141 cancerous samples and 89 controls.

**GSE7226-GPL2004**

Platform designation GPL2004,
comprising 102 samples from mentally retarded children and 213
controls from their unaffected siblings or parents. The first
2,000 SNPs were considered.

**GSE7226-GPL2005**

Platform designation GPL2005,
comprising 103 samples from mentally retarded children and 210
controls from their unaffected siblings or parents. The first 2,000
SNPs were considered.
