# Supplementary material for: Does replication groups scoring reduce false positive rate in SNP interaction discovery?
Source: BMC Genomics. 2010 Jan 22;11:58. doi: 10.1186/1471-2164-11-58 (PMC2823693; doi:10.1186/1471-2164-11-58)
Supplement: Additional file 3 — Performance graphs for differently sized subsets of GSE6754. Performance graphs for data subsets of 100, 200, 500, 1000, 2000, and 5000 samples drawn from GSE6754. [file 1471-2164-11-58-S3.ZIP › results_size.html]

Supplement to: Does replication groups scoring reduce false positive rate in
SNP interaction discovery?


## Performance graphs with differently sized subsets of GSE6754

Graphs present the dependency of false positive counts
given the number of selected best candidate interactions.
Curves closer to lower-right corner of the graph indicate better performance. The
axes are in logarithmic scale to emphasize the results for smaller
numbers of best candidates.

We used the GSE6754 data set, which describes families with two individuals
affected by autism spectrum disorders. Individuals were classified as
affected (2459 samples) or unaffected (3473 samples) and described with
around 10,000 SNPs each. Only the first 2,000 SNPs were used for the analysis.

### Curve legend

light gray - theoretically best and worst possible performance curves  
black solid - direct scoring  
black dashed - scoring with two replication groups  
black dotted - scoring with three replication groups

### data set size = 100 samples

### data set size = 200 samples

### data set size = 500 samples

### data set size = 1000 samples

### data set size = 2000 samples

### data set size = 5000 samples
