# Supplementary material for: Does replication groups scoring reduce false positive rate in SNP interaction discovery?
Source: BMC Genomics. 2010 Jan 22;11:58. doi: 10.1186/1471-2164-11-58 (PMC2823693; doi:10.1186/1471-2164-11-58)
Supplement: Additional file 4 — Source code and data sets. Source code and data sets needed to replicate the experiments. [file 1471-2164-11-58-S4.ZIP › add4/links.htm]

About the supplement  
Results  
 
